# Robust memory humoral immune response to SARS-CoV-2 in the tonsils of adults and children

**DOI:** 10.3389/fimmu.2023.1291534

**Published:** 2023-12-11

**Authors:** Tarfa A. Altorki, Rwaa H. Abdulal, Bandar A. Suliman, Talal M. Aljeraisi, Asem Alsharef, Wesam H. Abdulaal, Mohamed A. Alfaleh, Abdullah A. Algaissi, Rowa Y. Alhabbab, Hani Ozbak, Hamza Mohammed Eid, Yahya Ahmad Almutawif, Xuguang Li, Mohammed W. Al-Rabia, Qibo Zhang, Ahmed Bakur Mahmoud, Waleed H. Mahallawi, Anwar M. Hashem

**Affiliations:** ^1^ Vaccines and Immunotherapy Unit, King Fahd Medical Research Center, King Abdulaziz University, Jeddah, Saudi Arabia; ^2^ Department of Medical Laboratory Technology, Faculty of Applied Medical Sciences, King Abdulaziz University, Jeddah, Saudi Arabia; ^3^ Medical Laboratory Technology Department, College of Applied Medical Sciences, Taibah University, Madinah, Saudi Arabia; ^4^ Otorhinolaryngology, Head and Neck Surgery Department, Faculty of Medicine, Taibah University, Madinah, Saudi Arabia; ^5^ Department of Biochemistry, Faculty of Science, King Abdulaziz University, Jeddah, Saudi Arabia; ^6^ Department of Pharmaceutics, Faculty of Pharmacy, King Abdulaziz University, Jeddah, Saudi Arabia; ^7^ Department of Medical Laboratories Technology, College of Applied Medical Sciences, Jazan University, Jazan, Saudi Arabia; ^8^ Centre for Oncology and Regulatory Research, Biologic and Radiopharmaceutical Drugs Directorate, Health Products and Food Branch, Health Canada and World Health Organization Collaborating Center for Standardization and Evaluation of Biologicals, Ottawa, ON, Canada; ^9^ Department of Biochemistry, Microbiology and Immunology, Faculty of Medicine, University of Ottawa, Ottawa, ON, Canada; ^10^ Department of Clinical Microbiology and Immunology, Faculty of Medicine, King Abdulaziz University, Jeddah, Saudi Arabia; ^11^ Academic and Research Departments, Section of Immunology, School of Biosciences and Medicine University of Surrey, Surrey, United Kingdom; ^12^ Health and Life Research Center, Taibah University, Madinah, Saudi Arabia

**Keywords:** SARS-CoV-2, COVID-19, tonsils, humoral immunity, neutralizing antibodies, mucosal immunity

## Abstract

**Background:**

Adaptive humoral immunity against SARS-CoV-2 has mainly been evaluated in peripheral blood. Human secondary lymphoid tissues (such as tonsils) contain large numbers of plasma cells that secrete immunoglobulins at mucosal sites. Yet, the role of mucosal memory immunity induced by vaccines or natural infection against SARS-CoV-2 and its variants is not fully understood.

**Methods:**

Tonsillar mononuclear cells (TMNCs) from adults (n=10) and children (n=11) were isolated and stimulated using positive SARS-CoV-2 nasal swabs. We used endpoint enzyme-linked immunosorbent assays (ELISAs) for the measurement of anti-S1, -RBD, and -N IgG antibody levels and a pseudovirus microneutralization assay to assess neutralizing antibodies (nAbs) in paired serum and supernatants from stimulated TMNCs.

**Results:**

Strong systemic humoral response in previously SARS-CoV-2 infected and vaccinated adults and children was observed in accordance with the reported history of the participants. Interestingly, we found a significant increase in anti-RBD IgG (305 and 834 folds) and anti-S1 IgG (475 and 443 folds) in the stimulated TMNCs from adults and children, respectively, compared to unstimulated cells. Consistently, the stimulated TMNCs secreted higher levels of nAbs against the ancestral Wuhan strain and the Omicron BA.1 variant compared to unstimulated cells by several folds. This increase was seen in all participants including children with no known history of infection, suggesting that these participants might have been previously exposed to SARS-CoV-2 and that not all asymptomatic cases necessarily could be detected by serum antibodies. Furthermore, nAb levels against both strains were significantly correlated in adults (r=0.8788; *p* = 0.0008) and children (r = 0.7521; *p* = 0.0076), and they strongly correlated with S1 and RBD-specific IgG antibodies.

**Conclusion:**

Our results provide evidence for persistent mucosal humoral memory in tonsils from previously infected and/or vaccinated adults and children against recent and old variants upon re-exposure. They also highlight the importance of targeting mucosal sites with vaccines to help control infection at the primary sites and prevent potential breakthrough infections.

## Introduction

In late 2019, the novel severe acute respiratory syndrome-coronavirus-2 (SARS-CoV-2) emerged in Wuhan, China, and caused the global COVID-19 pandemic (1, 2). Since then, the virus has been evolving with emerging variants showing increasing capacity for antibody-evasion (3–5).

SARS-CoV-2 infection or vaccination elicits protective humoral and cellular immunity (6). Most current vaccines are based on the viral spike (S) protein inducing specific antibodies and T cell responses. The levels of neutralizing antibodies (nAbs) targeting the viral S protein, in particular the receptor-binding domain (RBD), have been shown to be associated with protection from infection, severe disease, or hospitalization (7). However, the majority of the initially approved and used vaccines were generated based on the S protein of the ancestral Wuhan strain, resulting in reduced protection against emerging SARS-CoV-2 variants including the Omicron variant and its subvariants (3–5). Additionally, several reports have suggested that such immune response is non-long-lasting (8, 9) and that variants such as Omicron are characterized by higher levels of immune escape and transmissibility compared to former variants, especially in individuals vaccinated with initially developed vaccines or infected with previously circulating variants (3–5). Nonetheless, recent versions of the vaccines are bivalent, targeting the ancestral Wuhan strain and the Omicron BA.1 variant, and should provide better protection against emerging variants (10, 11).

While some studies have investigated antibody response in oral and nasal secretions (12–14), the majority of previous reports have mainly focused on humoral and cellular immunity in the peripheral blood of vaccinated or infected individuals (14–16). On the other hand, very limited reports have studied SARS-CoV-2 infection or vaccine-induced immunity in lymphoid tissues of the upper respiratory tract, which are the primary sites of infection and replication of respiratory pathogens (17–19). Generally, long-lived memory B cells are the source of protective immune response upon antigen re-exposure following either infection or vaccination and are essential in maintaining the antiviral antibody response state against viral exposure (20, 21). Specifically, the development of adaptive immunity at mucosal sites and induction of tissue-resident memory B or T cells could help prevent and reduce infections at the original site, rather than simply limiting infection and progression of disease symptoms (22). Hence, understanding the role of local memory B cell responses in the upper mucosal respiratory sites in vaccinated and recovered individuals is important.

Human secondary lymphoid tissues (such as tonsils) contain large numbers of plasma cells that secrete immunoglobulins at mucosal sites (23). Thus, there is a need for vaccines capable of inducing strong and long-lasting humoral and cellular immune responses at local mucosal sites to provide protection against infection and to reduce transmission of respiratory pathogens (22). At present, approved COVID-19 vaccines are mostly developed for intramuscular (IM) immunization. Yet, they are ineffective in preventing viral transmission and infection in the upper respiratory tract due to insufficient induction of mucosal immunity. Numerous intranasal (IN) COVID-19 vaccines are in development and under clinical trials (24, 25), with two licensed IN vaccines BBV154 (Bharat) and AD5-nCOV (CanSino) in India and China, respectively (26–28). Such vaccines could be complementary to the current IM COVID-19 vaccines and could be employed in either homologous vaccination or heterologous booster approaches (22, 29, 30).

Here, we investigated the humoral memory immune responses of tonsillar-derived mononuclear cells (TMNCs) from adults and children against the ancestral Wuhan strain and the Omicron BA.1 variant upon ex vivo re-stimulation. We found that re-stimulation of TMNCs could elicit robust binding and nAbs in terms of titers and breadth against both strains in vaccinated and previously infected adults and children, suggesting that enhanced protection from infection could be achieved with better induction of mucosal immunity.

## Materials and methods

### Tonsillar and blood samples

In total, 21 patients who underwent elective tonsillectomy at the surgical ENT department at the Saudi German Hospital, Madinah, Saudi Arabia were recruited in the current study. The samples were collected from February to May 2022. Paired tonsils and serum samples were collected from all the individuals. Signed informed consent was obtained from all participants and parents of children. Only those who suffered from snoring or obstructive sleep apnea were included in this study. Patients with recurrent tonsillitis or any history of immune deficiency were excluded. Demographics, including age, sex, and history of vaccination, and previous COVID-19 infection data were collected from all participants. Ethical approval was obtained from the ethics committee of the General Directorate of Health Affairs, Ministry of Health, Madinah (IRB No. MLT 2022031).

### Isolation of TMNCs

**Figure 1:**
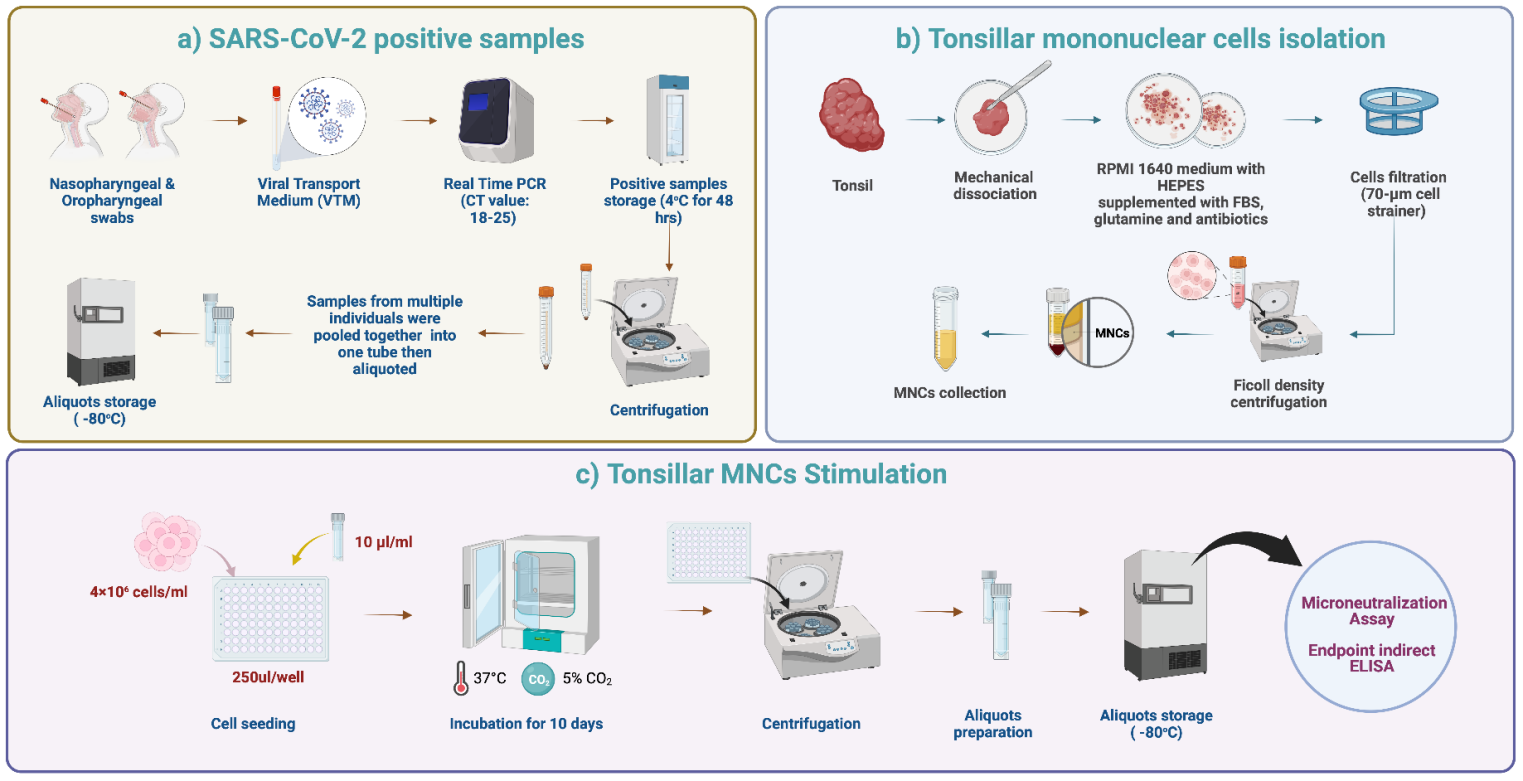
Tonsils processing and stimulation. **(A)** SARS-CoV-2 positive samples. Nasopharyngeal and oropharyngeal swabs were collected from each patient in viral transport media (VTM). Six laboratory-confirmed RT-PCR SARS-CoV-2 positive samples with Ct values of 18-25 were selected and pooled into one tube. The pooled samples were mixed well, aliquoted, and stored at -80°C to ensure that the same stimulant (viral load) was used for each tonsillar sample. **(B)** TMNCs isolation. Fresh tonsillar samples were processed within one hour after surgery. The tissues were cut into small pieces using a sterilized scalpel and then checked grossly. Then, the cell suspension was passed through a 70-μm sterile nylon mesh. TMNCs were separated by density gradient centrifugation using Ficoll-Paque (400 x g for 30 min). The cells were then washed twice using sterile phosphate-buffered saline (PBS) and resuspended in 5 ml complete RPMI 1640 medium for further processing and cell culture steps. **(C)** TMNCs stimulation. One aliquot of stored stimulation sample was thawed and 10 μl were added to 4 × 10^6^ cells/ml of TMNC suspension from each individual in 96 well plates. Negative control samples (unstimulated) were prepared by adding 10 μl of VTM only to a TMNC suspension from each individual. Following gentle mixing, 96-well cell culture plates were incubated in a 5% CO_2_ incubator at 37°C. Cell culture supernatants were collected after 10 days and stored at −80°C until assayed.

Cell suspensions were prepared using a modified protocol based on a previously described method (31). Briefly, fresh tonsillar samples were processed within 1 hour after surgery. The tissues were cut into small pieces using a sterilized scalpel and then checked macroscopically. The tissues were then kept in complete RPMI 1640 medium with HEPES, 10% fetal bovine serum, 2 mM glutamine, 100 U/ml penicillin, and 100 μg/ml streptomycin (Sigma-Aldrich) in a sterile petri dish to release the cells from the tonsillar tissue. The cell suspension was obtained by passing through a 70-μm sterile nylon mesh to eliminate tissue debris. TMNCs were separated with Ficoll-Paque (Premium GE Healthcare, UK) gradient centrifugation (400 g for 30 min). The cells were then washed twice using sterile phosphate-buffered saline (PBS) and then resuspended in 5 ml complete RPMI 1640 medium for further processing and cell culture steps (**Figure 1**).

### Stimulation of TMNCs

Laboratory-confirmed RT-PCR SARS-CoV-2 positive nasal swab samples with Ct values of 18-25 in viral transport media (VTM) were used for *ex-vivo* re-stimulation of the TMNCs. Specifically, six positive samples (300 μl each) were pooled into one tube, mixed very well, aliquoted, and stored at -80°C to ensure that the same stimulant (viral load) is used for each tonsillar sample. Upon collection and processing of each tonsillar sample and preparation of individual TMNC suspensions, one aliquot of stored stimulation sample was thawed and added in a volume of 10 μl to 4×10^6^ cells/ml of TMNC suspension from each individual. The ratio of stimulus/cell number was established following several optimization tests. Negative control samples (unstimulated) were prepared by adding 10 μl of VTM only to a TMNC suspension from each individual. Following gentle mixing and incubation for 5 hours, 250 μl of stimulated and unstimulated cell suspensions were transferred to a sterile 96-well cell culture plate, centrifuged and media was replaced with fresh complete RPMI and cultured in 5% CO_2_ incubator at 37°C. Cell culture supernatants were collected after 10 days and stored at −70°C until assayed (**Figure 1**). The Omicron BA.1 variant at the time of conducting the study was the dominant circulating variant worldwide and in Saudi Arabia and it accounted for 99.8% of all circulating variants (32).

### Microneutralization assay

The neutralization activity of serum samples and supernatants was determined using a recombinant vesicular stomatitis virus (rVSV)-based pseudovirus microneutralization assay as previously described (33, 34). In brief, rVSVs expressing codon-optimized full-length SARS-CoV-2 S protein from the ancestral Wuhan strain (rVSV-ΔG/SARS-2-S*-Wuhan pseudovirus) or the Omicron BA.1 variant (rVSV-ΔG/SARS-2-S*-BA.1 pseudovirus) were generated. BHK21/WI-2 cells transfected with pcDNA3.1 plasmid expressing S protein from either the ancestral Wuhan strain or the Omicron BA.1 variant were infected with rVSV-G/G∗-luciferase 24 hours after transfection, and the supernatant containing the rVSV pseudoviruses were collected after additional 24 hours. The collected pseudoviruses were titrated by measuring luciferase activity in infected Vero E6 cells. Titers were expressed as relative luciferase units (RLU). Then, a neutralization assay was performed by incubating two-fold serial dilutions of supernatant (unstimulated or stimulated) and heat-inactivated sera starting from a 1:10 dilution of supernatant samples and 1:20 dilution of serum samples (in duplicate) in DMEM with 5% FBS containing 5 × 10^4^ RLU of each pseudovirus for 1 hour at 37°C in a 5% CO_2_ incubator. The mixtures were then transferred to white 96-well plates containing confluent Vero E6 cells and incubated for 24 hours at 37°C in 5% CO_2_. After 24 hours, cells were lysed, luciferase activity was measured using Luciferase Assay System (Promega) according to the manufacturer’s instructions, and luminescence activity was measured using BioTek Synergy 2 microplate reader (BioTek, Winooski, VT). Each assay run included a cell-only control (CC) and a virus control (VC). The inhibition of luciferase activity by each dilution was determined as follows: 100 – [(average RLU from each dilution – average RLU from CC)/(average RLU from VC – average RLU from CC) × 100]. Then, neutralization titers were computed as half maximal inhibitory concentrations (IC_50_) using a four-parameter logistic (4PL) curve in GraphPad Prism V9 software (GraphPad Co., San Diego, CA).

### Endpoint indirect ELISA

Recombinant SARS-CoV-2 S1 subunit (amino acids 1–685), RBD (amino acids 319–541), and full-length nucleocapsid (N) proteins were purchased commercially (Sino Biological, China). An endpoint indirect enzyme-linked immunosorbent assay (ELISA) was performed for the detection of specific total IgG against S1, RBD, and N proteins as previously described (35). Briefly, recombinant S1, RBD, and N proteins were used to coat 96-well high-binding ELISA plates (Greiner Bio One, Monroe, NC) at 1 μg/ml in PBS with 50 μl per well. After overnight incubation at 4°C, plates were washed with PBS containing 0.05% tween-20 (PBS-T) and blocked with 5% skim milk in PBS-T buffer (blocking buffer) at 37°C for 1 hour. Plates were then washed and incubated with serially diluted supernatant (unstimulated or stimulated) and serum samples in blocking buffer starting from 1:20 or 1:100 for 1 hour at 37°C, respectively. Plates were then washed and incubated with goat anti-human IgG (Fcγ fragment specific) conjugated to HRP (Jackson ImmunoResearch, West Grove, PA) for 1 hour to detect total binding IgG. After washing, plates were incubated with TMB (3,3’,5,5’-tetramethylbenzidine) substrate (KPL, Gaithersburg, MD) at room temperature for 30 min, and the reaction was stopped by 0.16 M sulfuric acid. Optical density (OD) was measured at 450 nm using a BioTek Synergy 2 microplate reader (BioTek, Winooski, VT). Cutoff dilutions were 1:20 and 1:100 for supernatant and serum, respectively. In all assays, known human serum positive and negative control samples collected from recovered and previously COVID-19-infected individuals and pre-pandemic samples were used. Endpoint titers were expressed as the reciprocals of the highest dilution with an OD value above 0.1 which was used as the cutoff OD value. The titers were computed using a four-parameter logistic (4PL) curve in GraphPad Prism V9 software (GraphPad Co., San Diego, CA).

### Statistical analysis

Statistical analyses and graphical presentations were conducted with GraphPad Prism V9 software (GraphPad Co., San Diego, CA). Pearson’s correlation coefficient was used to assess the relationship between antibody titers. Data for continuous variables were presented as mean ± standard deviation (SD), while for categorical variables data were presented as frequency and percentage (%). Fisher’s exact test was used to assess the association between two categorical variables. Categorical variables were presented as proportions and percentages. Significance was reported as *, p < 0.05; **, p < 0.01; and ***, p < 0.001.

## Results

### Demographics

A total of 21 participants were included in this study of which 47.6% (n= 10) were adults and 52.4% (n= 11) were children. The mean age for children was 8.59 ± 3.95 years, while the mean age for adults was 25.0 ± 6.20 years. Of the sample, 62% (n= 13) were males, and 85.7% (n= 18) of the study sample indicated a history of previous infection. Specifically, all adults (10/10) reported previous infection compared to 8 out of the 11 children who had confirmed infection history. The mean time since last positive RT-PCR for children was 4.00 ± 7.79 weeks and the mean time since last positive RT-PCR for adults was 3.83 ± 1.91 weeks. The highest proportion of the study sample were vaccinated, 19.0% (n= 4; 2 adults and 2 children) received one dose, 33.3% (n= 7; 6 adults and 1 child) received two doses, and 9.50% (n= 2; all adults) received three doses of the vaccine. Most of the children (8/11) received no vaccine (**Table 1**).

**Table 1:** Demographics of participants.

No	Age	Sex	Vaccine doses	Previous infection	Time since +ve RT-PCR
Adults					
**A1**	22	M	2	Yes	4 weeks
**A2**	26	F	2	Yes	3 weeks
**A3**	39	M	3	Yes	3 weeks
**A4**	24	F	2	Yes	4 weeks
**A5**	30	F	3	Yes	2 weeks
**A6**	27	M	2	Yes	2 weeks
**A7**	20	M	1	Yes	4 weeks
**A8**	19	F	2	Yes	3 weeks
**A9**	25	M	2	Yes	8 weeks
**A10**	18	M	1	Yes	4 weeks
Children					
**C1**	14	F	2	Yes	2 weeks
**C2**	13	M	1	Yes	3 weeks
**C3**	6	F	NO	Yes	NA
**C4**	8	M	NO	Yes	NA
**C5**	11	M	NO	Yes	8 weeks
**C6**	7	F	NO	No	NA
**C7**	12	M	1	Yes	4 weeks
**C8**	4.5	M	NO	No	NA
**C9**	3	M	NO	Yes	NA
**C10**	4	M	NO	No	NA
**C11**	12	F	NO	Yes	24 weeks

NA, not available.

### Strong systemic humoral response in previously SARS-CoV-2 infected and vaccinated adults and children

**Figure 2:**
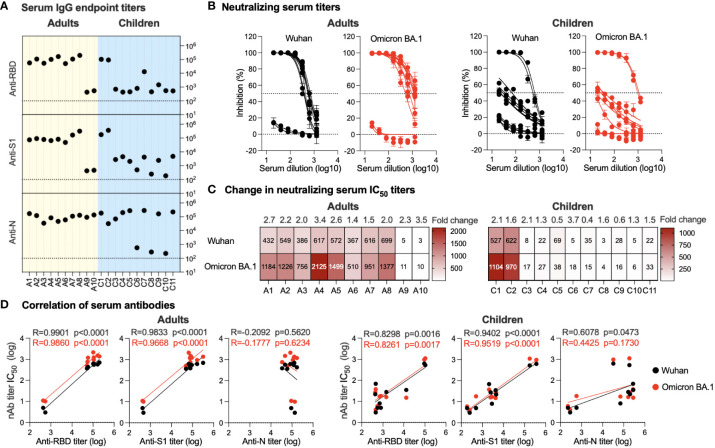
Serum antibody response. **(A)** Endpoint binding IgG titers in serum samples from adults and children were determined against SARS-CoV-2 N, S1, and RBD proteins. The dotted lines represent the cutoff of each assay. **(B)** Inhibitory activity of serum samples from adults and children against ancestral Wuhan strain and Omicron BA.1 variant pseudoviruses were determined. **(C)** Heatmap showing neutralizing antibodies IC_50_ obtained from analysis of panel **(B)** against ancestral Wuhan strain and Omicron BA.1 variant pseudoviruses. Fold change in IC_50_ is shown on top of the heatmap representing activity against the SARS-CoV-2 Omicron BA.1 variant relative to the ancestral Wuhan SARS-CoV-2 strain. **(D)** Correlation between neutralizing antibodies against the SARS-CoV-2 Omicron BA.1 variant and the ancestral Wuhan SARS-CoV-2 strain and binding IgG antibodies in serum is shown for anti-RBD, -S1, and -N antibodies from adults and children.

All serum samples were tested for the presence of total binding IgG specific to SARS-CoV-2 S1, RBD, and N proteins using an endpoint indirect ELISA. Overall, a robust immune response was observed in the serum of all adult participants despite their infection or vaccination status. A high-level anti-N IgG (range; 3.5x10^4^-1.7x10^5^) was found in all adults consistent with their previous history of infection (**Figure 2A**). Interestingly, only 8 individuals (A1-A8) showed strong anti-S1 (range; 5x10^4^-3x10^5^) and anti-RBD (range; 5x10^4^-2x10^5^) while two adult participants had low IgG titers against both S1 and RBD with a titer of ~5x10^2^ (**Figure 2A**). Similarly, while high serum nAb titers (IC_50_) were found in individuals A1-A8 against both the ancestral Wuhan SARS-CoV-2 strain and the Omicron BA.1 variant, very low levels of nAbs were seen in participants A9 and A10 (**Figures 2B, C**). The IC_50_ against the Omicron BA.1 variant (range; 510-2125) was higher by 1.4-3.4 folds than IC_50_ levels against the ancestral Wuhan strain (range; 366-698), as shown in **Figure 2C**. Of note, individuals A9 and A10 recently recovered from infection and received one or two doses of COVID-19 vaccine similar to other participants, suggesting that there could be individual variation in eliciting and maintaining humoral immune response (**Table 1**).

In children, anti-N IgG levels were found to be high in all the children’s samples (range; 3.210^4^-2.8x10^5^) except for three participants (C6, C8 and C10) with anti-N being <6x10^2^ (**Figure 2A**). While this is in accordance with their reported previous infection and vaccination status (**Table 1**), the anti-N IgG titers detected in C6, C8, and C10 were above the cutoff level and may indicate previous exposure to coronaviruses. On the other hand, the children’s serum showed marked variation in the IgG antibody response, ranging from strong anti-S1 levels (range; 1.7-3.7x10^5^) and anti-RBD (range; 9.4x10^4^ -1.1x10^5^) in the vaccinated children (C1 and C2) to weak anti-S1 response (range; 2.5x10^2^-4.7x10^3^) and anti-RBD (range; 4.5x10^2^-1.5x10^3^) in the non-vaccinated children (C3-C6 and C8-C11) (**Figure 2A**). Interestingly, individual C7, while having been previously infected and having received a single dose of the vaccine, had very low anti-S1 and -RBD IgG levels and were similar to non-vaccinated children (4.7x10^3^ and 1.3x10^4^, respectively). Consistent with the vaccination history and ELISA results, children C1 and C2 showed strong nAb titers with IC_50_ levels similar to those observed in adults against both the Omicron BA.1 variant and the ancestral Wuhan strain (**Figures 2B, C**). While for most previously infected non-vaccinated children (C4, C5, C9, and C11), except for child C3 who showed a weak nAb response, the response was higher than that seen in children with no previous history of infection (C6, C8, and C10) as shown in **Figures 2B, C**.

Both anti-S1 and anti-RBD IgG levels showed significant positive correlation with nAbs levels in both adults (p<0.0001 for anti-S1 and -RBD) and children (p<0.0001 for anti-S1 and p<0.002 for anti-RBD) against both Omicron BA.1 variant and ancestral Wuhan strain (**Figure 2D**). In contrast, no significant correlation was observed between anti-N IgG levels and nAbs except for a weak positive correlation between the children’s IgG and nAbs against the ancestral Wuhan strain (p=0.0463) (**Figure 2D**). Of note, anti-N IgG levels and nAbs in adults showed a non-significant negative correlation. The low or no correlation between nAbs levels and anti-N is expected as anti-N antibodies are not expected to have neutralizing activity like anti-S1 and -RBD antibodies.

### Strong humoral immunity in the tonsils of previously infected and vaccinated adults and children in response to SARS-CoV-2 stimulation

**Figure 3:**
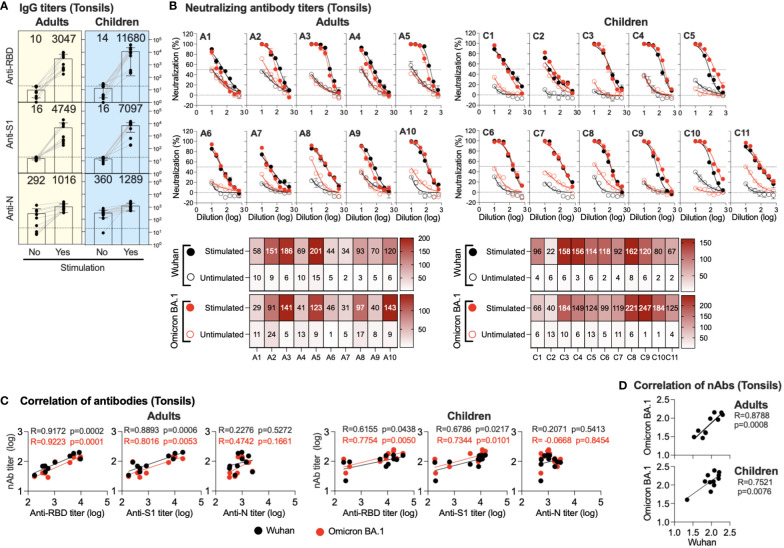
Induced antibodies in stimulated tonsils supernatants. Supernatants from stimulated or unstimulated mononuclear cells from tonsils were tested for binding and neutralizing antibodies. **(A)** Endpoint binding IgG titers in supernatants (unstimulated and stimulated) from adults and children were determined against SARS-CoV-2 N, S1, and RBD proteins. The mean titer is shown on top of each bar. **(B)** The inhibitory activity in supernatants (unstimulated and stimulated) from adults and children against ancestral Wuhan and Omicron BA.1 pseudoviruses was determined. Heatmap shows neutralizing antibodies (IC_50_) obtained from unstimulated and stimulated against ancestral Wuhan and Omicron BA.1 pseudoviruses. **(C)** Correlation between neutralizing antibody against the SARS-CoV-2 Omicron BA.1 variant and the ancestral Wuhan SARS-CoV-2 strain and binding IgG antibodies in the supernatants (unstimulated and stimulated) is shown from anti-RBD, -S1 and -N antibodies from adults and children. **(D)** Correlation between neutralizing antibody titers against the ancestral Wuhan SARS-CoV-2 strain vs the SARS-CoV-2 Omicron BA.1 variant is shown.

TMNCs were isolated from the tonsils of previously infected and/or vaccinated participants (adults=10 and children=11). The cells were then stimulated as indicated, followed by anti-S1, -RBD, and -N IgG titer measurement in stimulated and unstimulated cells using endpoint ELISA. As shown in **Figure 3A**, significant IgG induction was seen in all stimulated TMNCs compared to unstimulated tonsils against all viral components. Specifically, we found a marked increase in anti-RBD IgG (305 and 834 folds) and anti-S1 IgG (475 and 443 folds) in adults and children, respectively. On the other hand, anti-N was slightly induced in all stimulated TMNCs compared to unstimulated cells with only a 3.5- and 3.6-fold increase in adults and children, respectively. Interestingly, the same level of induced IgG response was also found in the children with no known history of infection (C6, C8, and C10) (**Table 1**), suggesting that these participants might have been indeed previously exposed to SARS-CoV-2 or other coronaviruses (36).

Similarly, stimulation of the TMNCs resulted in the secretion of nAbs against both ancestral Wuhan strain and Omicron BA.1 variant pseudoviruses despite the reported previous infection or vaccination history of all participants (**Figure 3B**). Although the IC_50_ levels of nAbs against the ancestral Wuhan strain and the Omicron BA.1 variant in the supernatant of stimulated tonsillar cells in adults and children were low, they were higher than the levels observed in unstimulated cells by several folds. These data confirm the binding IgG data and indicate that all these individuals had been exposed to SARS-CoV-2 previously.

While no significant correlation was found between anti-N IgG levels and nAbs, induced anti-S1 IgG levels significantly correlated with nAbs levels in both adults (r = 0.8893; *p* =0.0006 and r = 0.8016; *p* =0.0053) and children (r = 0.6786; *p* =0.0217 and r = 0.7344; *p* =0.0101) against both the Omicron BA.1 variant and the ancestral Wuhan strain, respectively (**Figure 3C**). Similarly, anti-RBD IgG levels showed a significant correlation with nAbs levels against both the Omicron BA.1 variant (r = 0.9223; *p* = 0.0001 and r = 0.7754; *p* =0.0050) and the ancestral Wuhan strain (r = 0.9172; *p* = 0.0002 and r = 0.6155; *p* =0.0438) in both adults and children, respectively (**Figure 3C**). Furthermore, nAb levels against the Omicron BA.1 variant and the ancestral Wuhan strain were significantly correlated in adults (r=0.8788; *p* = 0.0008) and children (r = 0.7521; *p* = 0.0076) as shown in **Figure 3D**.

### Correlation between serum and tonsillar antibody responses

**Figure 4:**
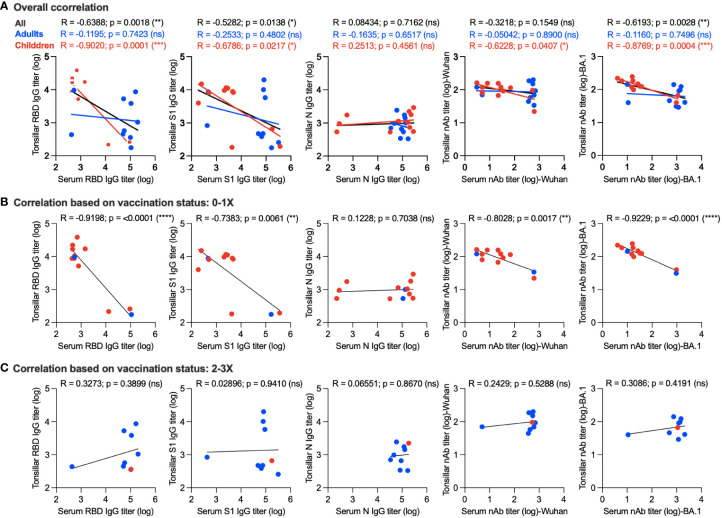
Correlation between memory humoral immune response in serum and stimulated tonsillar cells. **(A)** Overall correlation between antibody response in serum and stimulated tonsillar cells. Correlation between systemic and local memory humoral immune response based on the vaccination status in unvaccinated individuals and those who received a single dose only **(B)** compared to participants who received two or three doses of the vaccine **(C)**. *, p < 0.05; **, p < 0.01; ***, p < 0.001; and ****, p < 0.0001. ns: non significant.

We further tried to gain insight into the correlation between memory humoral immune response in the supernatant of stimulated TMNCs and serum. Interestingly, we found a significant negative correlation between serum and tonsillar memory anti-RBD IgG (r = -0.6388; *p* = 0.0018), anti-S1 IgG (r = -0.5282; *p* = 0.0138), and nAbs against the BA.1 variant (r = -0.6193; *p* = 0.0028) as shown in **Figure 4A**. By examining the correlation in adults and children, it was clear that a significant inverse correlation was only found in children but not adults in anti-RBD IgG (r = -0.9020; *p* = 0.0001), anti-S1 IgG (r = -0.6786; *p* = 0.0217), nAbs against the Wuhan strain (r = -0.6228; *p* = 0.0407), and nAbs against BA.1 variant (r = -0.8769; *p* = 0.0004) (**Figure 4A**), suggesting that the age of the participants could be a factor leading to such negative correlation.

Next, we determined the correlation between systemic and local memory humoral immune response based on the vaccination status in unvaccinated individuals and those who received a single dose only (10 children and 2 adults) (**Figure 4B**) compared to participants who received two or three doses of the vaccine (1 child and 8 adults) (**Figure 4C**). As shown in **Figure 4B**, a significant inverse correlation was found in anti-RBD IgG (r = -0.9198; *p* = <0.0001), anti-S1 IgG (r = -0.7383; *p* = 0.0061), nAbs against the Wuhan strain (r = -0.8028; *p* = 0.0017), and nAbs against the BA.1 variant (r = -0.9229; *p* = <0.0001) of those who received no or one dose. This correlation was significantly higher than that observed based on age (**Figure 4A**). In contrast, we found a positive correlation between antibody responses in the serum and stimulated tonsillar cells in those who received two doses or more as one should expect, however, this correlation did not reach significant levels (**Figure 4C**). Taken together, these data suggest that not only age but also the number of doses could affect the level of memory antibody response in mucosal lymphoid tissues and its correlation with serum levels.

## Discussion

Durable antibody-mediated immune defense depends on the development of high-affinity memory cells and long-lived plasma cells that can rapidly respond to secondary antigen exposure. While serum circulating antibodies are crucial in maintaining durable humoral immunity (37, 38), antibodies secreted by B cells in mucosal secondary lymphoid organs such as tonsils are critical in responding to respiratory pathogens at the primary site of infection. Tonsillar memory B cells can rapidly differentiate into antibody-secreting cells after pathogen invasion compared to the slower activation and differentiation of circulating memory B cells in peripheral blood. Additionally, *in vitro* stimulation of tonsillar memory B cells can lead to the production of a higher amount of antibodies compared to their peripheral counterpart populations (21). Furthermore, the tonsils could act as upper airway sentinels and they are an efficient site for antigen uptake and induction of local specific humoral immune responses.

While it has been shown that SARS-CoV-2 infection and vaccination can induce long-lasting humoral and cellular immune responses in children and adults, this was mostly determined by assessing immunity in peripheral blood. On the other hand, little is known about virus-specific adaptive immunity in lymphoid tissues of the upper respiratory tract. Few previous reports have investigated such responses in mucosal sites of the upper respiratory tract (17–19). These studies have shown that SARS-CoV-2 infection can induce robust virus-specific memory responses and germinal center B cells, CD4^+^ and CD8^+^ T cells, and T follicular helper cells in the tonsils and lung-draining lymph nodes of children and adults that could last up to 7-10 months post-infection (17–19). The B cells in the tonsils were mostly immunoglobulin class-switched, somatically hypermutated, and affinity matured and had distinct tissue-resident profiles compared to their counterpart in circulation (18).

In this study, the detection of circulating anti-N IgG in all adults and children provided serological evidence of their previous infection despite their reported history. Nonetheless, the low levels of anti-N IgG do not necessarily indicate a history of no infection as antibodies could wane over time (35, 39). Notably, 2 out of the 10 adults showed weak circulating anti-S1 and anti-RBD IgG and nAb responses compared to the rest of the group despite their previous history of infection and receiving one or two doses of COVID-19 vaccine. Higher variability of anti-S1 and anti-RBD binding IgG antibodies and nAbs were seen in children ranging from robust responses in vaccinated subjects (C1 and C2) to weak antibodies in non-vaccinated children (C3-C6 and C8-C11). Although antibodies can wane over time, these results highlight the heterogeneity of the humoral immune responses, especially in children in response to infection or vaccines (18, 19), and underscore the need to better understand SARS-CoV-2 immunity to help control viral spread and infection and develop better countermeasures.

Interestingly, when the TMNCs were stimulated, we observed a marked increase in anti-RBD and anti-S1 IgG in adults and children with lower induction of anti-N in the stimulated TMNCs. This increase was seen in all participants including the children with no known history of infection (C6, C8, and C10), suggesting that these participants might have been previously exposed to SARS-CoV-2. Similarly, the IC_50_ levels of nAbs against both the ancestral Wuhan strain and the Omicron BA.1 variant from the supernatant of the simulated TMNCs were higher than the levels observed in unstimulated cells by several folds. These data confirm the binding IgG data and suggest that all these individuals had been exposed to SARS-CoV-2 previously. Furthermore, these results highlight the magnitude of SARS-CoV-2 spread and the underappreciated number of undiagnosed asymptomatic cases that might not necessarily be detected by serum antibodies.

The observation of cross-neutralizing antibodies against the Omicron BA.1 variant and the ancestral Wuhan strain is most likely due to the shared epitopes between the two variants. Indeed, the significant increase in binding IgG levels from stimulated TMNCs against S1 and RBD from the ancestral Wuhan strain which we used in our ELISA indicates that there are shared epitopes in these neutralization-rich epitope regions. While there is no information about the variants that infected the participants in our study, it is assumed that they were infected with an Omicron variant as it was the dominant circulating variant worldwide and in Saudi Arabia in late 2021 and early 2022, accounting for 99.8% of all circulating variants (32). Thus, our results, especially in adults, are aligned with previous reports that demonstrated that Omicron infection of vaccine-experienced individuals mediates broadly neutralizing activity against BA.1, BA.2, and several previous SARS-CoV-2 variants, including the ancestral Wuhan strain (40). This is because BA.1 breakthrough infection could induce a strong recall response, likely through expanding memory B cells against epitopes shared broadly amongst variants, rather than inducing BA.1-specific B cells (40).

It is now well-accepted that infection and vaccination can elicit circulating humoral and cellular immunity against SARS-CoV-2 that lasts for 1-2 years, and that the level of nAbs in peripheral blood correlates with protection. However, less is known about memory mucosal responses, especially in secondary lymphoid organs such as tonsils and adenoids. Respiratory viruses colonize the mucosal surfaces prior to systemic infection making it very crucial to target mucosal immunity. While parenterally directed vaccines would provoke a strong systemic immunity, they normally elicit a frail mucosal immunity compared to natural infection which induces a strong mucosal humoral response (30). Additionally, it was shown that a combination of systemic and mucosal vaccination could elicit stronger mucosal nAbs compared to systemic or mucosal vaccination alone (22, 24, 30). Hence, it would be ideal to design and develop booster mucosal vaccine candidates as they offer better protection at the primary site of infection and help prevent potential breakthrough infection by Omicron variants and future VOCs (41, 42).

Interestingly, we found significant negative correlations between mucosal and systemic anti-S1, anti-RBD, and nAb responses. This inverse relationship was mostly evident in samples from children compared to adults and those who received no or one vaccine dose compared to those who received two or three vaccine doses. In contrast, we found positive correlations between mucosal and systemic antibody titers among all the participants who received two or three vaccine doses; however, this correlation did not reach the significance level. These findings suggest that age as well as vaccine doses could affect the levels of mucosal immunity in individuals receiving IM vaccines. Additionally, they may also indicate that the induction of sufficient or potent memory B cell responses in mucosal sites may require better immunization approaches such as IN vaccines. Previous studies have shown a positive correlation between serum as well as secreted mucosal antibody levels and number of COVID-19 vaccine doses (43, 44) in addition to other factors such as age and gender (45). Therefore, our data have to be interpreted with caution given the small sample size in the study and the potential influence of gender, age, and number of vaccine doses received. Nonetheless, our report highlights the importance of a better understanding of the mucosal memory immune responses in different populations based on age, gender, type of vaccines or doses received, and previous infection history.

While the small sample size is a limitation in our study due to the difficulties of collecting a large number of such samples, other limitations include the lack of samples from individuals with a long history of infection with earlier circulating variants to evaluate cross-reactivity against recent variants. The lack of control samples from before 2020 is also a limitation that could have informed us about the potential cross-reactivity between other coronaviruses and SARS-CoV-2. In addition, there was no information about the identity of the variant that infected the participants in our study although the Omicron variant was the dominant circulating variant worldwide and in Saudi Arabia and it accounted for 99.8% of all circulating variants at the time of conducting the study (32). Missing information on the type of vaccines administered and the sequence and time of infection in subjects are other limitations in the study. Testing longitudinal samples could help map the duration of mucosal immunity in TMNCs. Furthermore, we believe future studies should investigate T and B cell responses in Peripheral blood mononuclear cells (PBMCs) and TMNCs as well as the different antibody isotypes in serum and tonsils to better understand the correlation between systemic and mucosal responses to infection and/or vaccination.

Our results provide insights into the nature of the vaccine- and/or infection-induced humoral memory immune response in the tonsils of adults and children, and how such response could provide protection against SARS-CoV-2 and its consequences upon re-exposure. Additionally, the robust induction and breadth of binding and neutralizing antibodies from re-stimulated TMNCs suggest that better protection against SARS-CoV-2 or other respiratory viruses could be achieved with better induction of mucosal immunity. Finally, we believe that using pharyngeal and tonsillar tissues could help us better understand the immune response at the primary site of infection response to vaccine and infection not only in healthy individuals but also in those with underlying conditions and patients with varied disease outcomes.

## Data availability statement

The original contributions presented in the study are included in the article/supplementary material. Further inquiries can be directed to the corresponding authors.

## Ethics statement

The studies involving humans were approved by committee of the General Directorate of Health Affairs, Ministry of Health, Madinah (IRB No. MLT 2022031). The studies were conducted in accordance with the local legislation and institutional requirements. Written informed consent for participation in this study was provided by the participants’ legal guardians/next of kin.

## Author contributions

TA: Data curation, Formal analysis, Investigation, Methodology, Writing – original draft. RA: Data curation, Methodology, Writing – original draft. BS: Data curation, Writing – review & editing. TMA: Formal analysis, Writing – review & editing. AA: Data curation, Visualization, Writing – original draft. WA: Supervision, Writing – review & editing. MA: Data curation, Methodology, Writing – review & editing. AAA: Supervision, Writing – review & editing. RYA: Data curation, Formal analysis, Writing – review & editing. HO: Investigation, Project administration, Writing – review & editing. HE: Formal analysis, Writing – review & editing. YA: Software, Validation, Writing – review & editing. XL: Resources, Writing – review & editing. MWA: Methodology, Validation, Writing – review & editing. QZ: Supervision, Writing – review & editing. AM: Formal analysis, Writing – review & editing, Writing – original draft. WM: Data curation, Supervision, Writing – review & editing. AH: Supervision, Writing – review & editing.
